# SinR Controls Enterotoxin Expression in *Bacillus thuringiensis* Biofilms

**DOI:** 10.1371/journal.pone.0087532

**Published:** 2014-01-31

**Authors:** Annette Fagerlund, Thomas Dubois, Ole-Andreas Økstad, Emilie Verplaetse, Nathalie Gilois, Imène Bennaceur, Stéphane Perchat, Myriam Gominet, Stéphane Aymerich, Anne-Brit Kolstø, Didier Lereclus, Michel Gohar

**Affiliations:** 1 Laboratory for Microbial Dynamics (LaMDa) and Department of Pharmaceutical Biosciences, School of Pharmacy, University of Oslo, Oslo, Norway; 2 Micalis, INRA (UMR1319), Domaine de Vilvert, Jouy-en-Josas, France; 3 Micalis, AgroParistech (UMR1319), Domaine de Vilvert, Jouy-en-Josas, France; 4 Institut Pasteur, CNRS URA 2172, Unité de Biologie des Bactéries Pathogènes à Gram positif, Paris, France; The University of Texas-Houston Medical School, United States of America

## Abstract

The entomopathogen *Bacillus thuringiensis* produces dense biofilms under various conditions. Here, we report that the transition phase regulators Spo0A, AbrB and SinR control biofilm formation and swimming motility in *B. thuringiensis*, just as they control biofilm formation and swarming motility in the closely related saprophyte species *B. subtilis*. However, microarray analysis indicated that in *B. thuringiensis*, in contrast to *B. subtilis*, SinR does not control an *eps* operon involved in exopolysaccharides production, but regulates genes involved in the biosynthesis of the lipopeptide kurstakin. This lipopeptide is required for biofilm formation and was previously shown to be important for survival in the host cadaver (necrotrophism). Microarray analysis also revealed that the SinR regulon contains genes coding for the Hbl enterotoxin. Transcriptional fusion assays, Western blots and hemolysis assays confirmed that SinR controls Hbl expression, together with PlcR, the main virulence regulator in *B. thuringiensis*. We show that Hbl is expressed in a sustained way in a small subpopulation of the biofilm, whereas almost all the planktonic population transiently expresses Hbl. The gene coding for SinI, an antagonist of SinR, is expressed in the same biofilm subpopulation as *hbl*, suggesting that *hbl* transcription heterogeneity is SinI-dependent. *B. thuringiensis* and *B. cereus* are enteric bacteria which possibly form biofilms lining the host intestinal epithelium. Toxins produced in biofilms could therefore be delivered directly to the target tissue.

## Introduction

*Bacillus subtilis* and pathogenic bacteria of the *Bacillus cereus* group (*B. cereus, B. thuringiensis* and *B. anthracis*) are all Gram-positive, flagellated, sporulating, and aerobic bacteria clustering closely in the phylogenetic tree of the *Bacillus* genus ([1]; http://www.patricbrc.org/portal/portal/patric/Phylogeny?cType=taxon&cId=1386). They share a large number of transcriptional factors, including the sporulation regulator Spo0A, the stress response sigma factor σ^B^, and the phase-transition regulators SinI, SinR, CodY and AbrB [2]. However, there are also important differences in the regulatory pathways between *B. subtilis* and *B. cereus sensu lato*. For example, the stress regulator σ^B^ is not activated in the same way in these species [3]; the two-component system DegU/DegS and the motility regulator SigD are absent from *B. cereus sensu lato* [2]; the virulence regulator PlcR, which promotes the transcription of numerous genes for extracellular enzymes and toxins and plays an important role in *B. cereus* and *B. thuringiensis* physiology [4]–[6], is absent from *B. subtilis*. These differences may well be the consequences of adaptation of these species to different ecosystems. *B. subtilis* is a saprophyte living on soil organic matter, whereas, *B. thuringiensis* is an entomopathogenic bacterium, genetically closely related to the human opportunistic pathogen *B. cereus* [7], [8], and to the human pathogen *B. anthracis* [9].

Both *B. subtilis* and *B. thuringiensis*, or *B. cereus*, can form biofilms at air-liquid interfaces. Biofilms are widely found structures in which microorganisms are protected against various stresses, allowing them to persist in adverse environmental conditions. The regulatory pathways in *B. subtilis* leading either to biofilm formation or to sporulation share the same initial steps. The transcriptional regulator Spo0A controls entry into sporulation [10], and is required for biofilm formation [11]. Spo0A represses *abrB* transcription [12] and promotes the transcription of *sinI* [13], the product of which is the SinR antagonist SinI. Both AbrB and SinR repress the two polycistronic operons *tapA-sipW-tasA* and *epsA-O* [14], [15]. The 15-gene *epsA-O* operon is involved in the biosynthesis of the exopolysaccharide component of the biofilm matrix [16] and the three-gene *tapA-sipW-tasA* operon is involved in the production of the protein component of the biofilm matrix [17], [18]. An inhibitor of flagellar motility is encoded by the *epsE* gene which is part of the *epsA-O* operon [19]. Therefore, deletion of *sinR* from *B. subtilis* results in an overproduction of biofilm and in impaired motility, whereas deletion of *sinI* results in the reverse phenotype. A paralogue of SinR, SlrR, is also involved in the control of biofilm formation and motility through its interaction with SinR [20], [21].

How biofilm formation is regulated in *B. thuringiensis* or in *B. cereus* is still unknown. In *B. anthracis*, SinR strongly represses the *sipW-tasA* operon [22], but the effect of *sinR* deletion on biofilm formation has not been studied. The quorum sensing molecule AI-2 is produced by *B. cereus* and inhibits biofilm formation when added exogenously [23], and the transcriptional regulators PlcR and CodY affect biofilm formation in the *B. cereus* reference strain ATCC 14579 [24]–[26]. PlcR is the main virulence regulator in *B. cereus* [6] and CodY, which represses the biosynthesis of branched amino-acids, might also be involved in the pathogenicity of *B. cereus* [26]–[29]. These findings suggest a connection between biofilm formation and virulence in this species. Here we report an investigation of the roles of Spo0A, AbrB and SinI/SinR in biofilm formation in the *B. thuringiensis* strain 407, which produces dense pellicles at the air-liquid interface. We found that SinI/SinR had a large effect on biofilm formation. We therefore analyzed the *B. thuringiensis sinR* regulon, which was found to include the *sipW-tasA* operon, but surprisingly no *eps* operon. SinR was also found to control the transcription of genes required for the production of lipopeptides previously shown to be involved in the bacterial survival in the host [30], and the transcription of enterotoxin genes.

## Materials and Methods

### Strains

Strains used in this study are listed in table S1. The acrystalliferous *B. thuringiensis* strain 407 Cry^−^ (genome sequence at NCBI : NZ_CM000747) is genetically closely related to the *B. cereus* reference strain ATCC 14579 [31]; however, strain 407 forms thick biofilms, while ATCC 14579 is a poor biofilm producer. Locus tags listed below follow the annotations of the sequenced ATCC 14579 strain genome (NC_004622), and the corresponding locus tags in the sequenced 407 strain genome are given table S2.

### Strain construction

The *sinI-sinR* locus in strain 407 was disrupted by insertion of a tetracycline resistance (TetR) cassette. A 937 bp HindIII-EcoRI fragment and a 758 bp *Xba*I–*Bam*HI fragment, corresponding to the chromosomal DNA regions upstream and downstream of the *sin* genes locus, respectively, were generated by PCR using the primer pairs Sin1-Sin14 and Sin17-Sin18 (table 1). The TetR cassette was purified from pHTS2 [32] as a 1.5 kb *Xba*I-*Eco*RI fragment. The amplifed DNA fragments and the TetR cassette were inserted between the *Hin*dIII and *Bam*HI sites of pRN5101 [33]. The resulting plasmid was used to transform the 407 wild type strain by electroporation, and the *sin* locus was deleted and replaced with the TetR cassette *via* allelic exchange by homologous recombination, as previously described [33]. The resulting mutant strain was designated *sinI-sinR*. The same procedure was used to disrupt *sinI*, *sinR* (BC1283 and BC1282, respectively) and *abrB* (BC0042): *sinI* and *sinR* were each disrupted with the tetracycline resistance cassette; and *abrB* with a kanamycin resistance cassette (a 1365 bp *Xba*I-*Pst*I fragment of pDG783 [34]). The resulting mutant strains were designated *sinI*, *sinR*, and *abrB*. The primer pairs used for these disruptions are listed Table 1.

**Table 1:** Primers used in this study.

Primer	sequence^a^	restriction site
Sin1	CCCAAGCTTTACCAGAAACTGTTAACC	*Hin*dIII
Sin2	CCGGAATTCGGCAAGTTCAGTTAATGA	*Eco*RI
Sin3	CGCTCAGATGCTGGTATCGCC	*Xba*I
Sin4	CGGGATCCTGTATACGAAACAACTTTAGC	*Bam*HI
Sin11	CGCTCAGATGCTGGTATCGCC	*Xba*I
Sin12	CCGGAATTCAAGTATTAAATCAATCCATTC	*Eco*RI
Sin14	CCGGAATTCCCTCTATGGAAATTATAAATTG	*Eco*RI
Sin17	GCTCTAGATGCAATGAACTCTGGTGTCTCC	*Xba*I
Sin18	CGCGGATCCTAGGGGGAATTGATTGTGAGTC	*Bam*HI
AbrB1FW	CCCAAGCTTGGGCTGCTAAATCTTCTAATCCCG	*Hin*dIII
AbrB1RV	GCTCTAGAGCCAATCATTTACATTTCCGTC	*Xb*aI
AbrB2FW	AAACTGCAGCGACATGATAGATTTGATATACATC	*Pst*I
AbrB2RV	CGCGGATCCAAAATATGTAGAGACCCACGAT	*Bam*HI
Hbl_pHT304_FW	CCAAGCTTGATATTAGGATGTTTTGTGA	*Hin*dIII
Hbl_pHT304_RV	CGGGATCCTTTACCATTGTTTTTATAAC	*Bam*HI
Yfp-F	GGGGTACCACATAAGGAGGAACTACTATGAGTAAAGGAGAAGAAC	*Kpn*I
Yfp-R	CGGAATTCTTATTTGTATAGTTCATCCATGC	*Eco*RI
Apha3_pHT304_FW	CCAAGCTTGATAAACCCAGCGAACCATTTGAGG	*Hin*dIII
Apha3_pHT304_RV	CGGGATCCCCGGTGATATTCTCATTTTAGCC	*Bam*HI
psinI-Rev BamHI	CGGGATCCCTAATTATCGGTCATAATTGC	*BamHI*
phbl-sinI-SOE-Fwd	aacatcctaatatTCTAGAatatttagtcatatcatg	none
phbl-sinI-SOE-Rev	atgactaaatatTCTAGAatattaggatgttttgtg	none
Hbl_pHT304_RV	CGGGATCCTTTACCATTGTTTTTATAAC	*BamHI*

The primers were used to delete the *sinI*, *sinR*, *sinI-sinR* and *abrB* genes from the strain 407, or to create transcriptional fusions between the promoters of *hbl* or of *sinI* and *lacZ*, *yfp* or *mcherry* on the pHT304-18 plasmid.

a : underlined sequences indicate the location of restriction sites, and lower case letters indicate overlapping sequences complementary to P*hbl* (not underlined) or to P*sinI* (underlined).

The antibiotic concentrations used for bacterial selection were as follows: 100 µg.ml^−1^ of ampicillin for *E. coli*; and 10 µg.ml^−1^ of erythromycin, 200 µg.ml^−1^ of kanamycin and 10 µg.ml^−1^ tetracycline for *B. thuringiensis*.

### Transcriptional fusions and beta-galactosidase assays

Expression of the *hbl* operon in the 407 wild type and mutant strains was monitored using a transcriptional fusion between the *hbl* promoter region and *lacZ*. The DNA sequence containing the *hbl* promoter was amplified using primers Hbl_pHT304_FW and Hbl_pHT304_RV (table 1) and inserted into pHT304-18Z [35], to give pHT304-18ΩP*_hbl_'-lacZ*. The same DNA sequence was inserted into pHT304-18YFP, resulting in pHT304-18ΩP*_hbl_'-yfp*. The DNA sequence containing the *apha3* promoter was amplified from pDG783 [34] with primers Apha3_pHT304_FW and Apha3_pHT304_RV (table 1) and inserted into pHT304-18YFP, resulting in pHT304-18ΩP*_apha3_'-yfp*. The plasmid pHT304-18YFP was constructed by insertion, between the sites *Eco*RI and *Kpn*I of pHT304-18 [36], of the *yfp* gene amplified from pKL183 [37] using the primer pair Yfp-F and Yfp-R (table 1). The plasmid pHT304-18ΩP*_hbl_'yfp*-P*_sinI_'mCherry* used to monitor simultaneously, in the same cell, *hbl* and *sinI* expressions, was constructed as follows. DNA fragments containing the promoter of *sinI* and *hbl* were amplified by PCR using the primers pairs phbl-sinI-SOE-Fwd/psinI-Rev BamHI and phbl-sinI-SOE-Rev/Hbl_pHT304_RV, respectively (table 1). These fragments were annealed to each other through complementary overlapping sequences introduced in primers phbl-sinI-SOE-Fwd and phbl-sinI-SOE-Rev. A single DNA fragment containing the promoter elements of *sinI* and *hbl* in opposite directions was then generated by PCR amplification with the primers psinI-Rev BamHI and Hbl_pHT304_RV. The resulting 1225 bp fragment was digested with *Bam*HI and cloned in the promoter free pHT304-18Ω*yfp-mCherry* digested with the same enzyme.

Electroporation was used to transfer pHT304-18ΩP*_hbl_'-lacZ*, pHT304-18ΩP*_hbl_'-yfp*, pHT304-18ΩP*_apha3_'-yfp* and pHT304-18ΩP*_hbl_'yfp*-P*_sinI_'mCherry* into 407 wild type or into 407 *sinI* strains. Beta-galactosidase specific activities were measured as described previously, and are expressed in units of beta-galactosidase *per* milligram of protein [38]. Beta-galactosidase was extracted from cells in biofilm obtained in glass tube assays (see below), and from planktonic cultures grown in LB medium at 30°C and 175 rpm. Three replicates were performed for each assay.

### Biofilm assays

The ability of 407 wild type and mutant strains to form biofilms in PVC (polyvinylchloride) microtiter plates was measured as described previously [23]. The method used to obtain biofilm in glass tubes was similar to that used in microtiter plates, with the following differences. Sterilized 6 ml glass tubes were inoculated with 2 ml of the cultures diluted to an OD_600_ of 0.01, and incubated for 48 h. The 2 ml culture medium was then removed using a Pasteur pipette and the OD_600_ of the ring and of the pellicle, thoroughly vortexed in 1 ml PBS, was measured. The microtiter plate and glass tube assays measure different parts of the biofilm: in the microtiter plate assay, the pellicle is lost during the staining procedure and the biofilm mass determined corresponds to the ring adhering at the air-liquid-solid interface. In the glass tube assay, the entire biofilm is recovered. Biofilms were formed in glass tubes for binocular microscopy observation, for beta-galactosidase assays, for fluorescence microscopy observation or for flow cytometry experiments.

### Swimming assays

The swimming ability of the 407 mutant strains was determined on LB 0.3% agar plates. Strains were grown in LB medium at 37°C with shaking at 175 rpm until the culture reached an OD_600_ of 1. A 5 µl drop of each culture was spotted on an agar plate and incubated at 30°C for 12 hours. Each experiment was repeated four times.

### Microarray analysis

Cells used for microarray analysis were grown in bactopeptone medium at 30°C and 250 rpm. Samples were collected 2 hours after entry into stationary phase (t2). Entry into stationary phase (t0) was determined as the breakpoint of the growth curve, i.e. the time point when the slope of the growth curve starts to decrease, which usually occurs in the 407 strain around OD 2.5 in these culture conditions. RNA isolation and cDNA synthesis, labeling and purification were performed as described previously [6]. Microarray slides were printed at the microarray core facility of the Norwegian University of Science and Technology (NTNU). Design, printing, prehybridization, hybridization and scanning of the slides and analysis of the data was as previously described [6]. The microarray experiments were based on four slides, all being true biological replicates. Genes with false discovery rate corrected p-values<0.05, and for which differential expression between the sinR-negative and sinR-positive strains was at least two-fold, were considered to be repressed (fold change FC>2.0) or induced (FC<0.5) by the deleted gene.

The microarrays used contain 70-mer oligonucleotide probes designed to detect open reading frames (ORFs) in *B. anthracis* strain Ames, *B. anthracis* strain A2012, *B. cereus* strain ATCC 14579, and *B. cereus* strain ATCC 10987 [39]. Only probes with 93% identity or greater to a transcript/feature sequence of 407 were included in the analysis. Of the predicted genes of the 407 genome, 1719 did not have corresponding probes on the array. However, among these genes, 1165 were annotated as hypotheticals (68%), and 761 were on contigs 00213 and 00060, which have later been shown to be plasmid-borne [40]. Microarray data are available in the ArrayExpress database (www.ebi.ac.uk/arrayexpress) under accession number E-MTAB-1806.

### Hemolysis assays

The various strains were grown in LB medium at 37°C until they reached an OD_600_ of 1. The hemolytic activity of the strains was assayed by loading a 5 µl drop of each culture on sheep blood agar plates. The agar plates were incubated overnight at 30°C and then scanned: the area of hemolysis and the colony size were determined from the scans with ImageJ software [41]. To minimize differences due to variations in growth rates, a hemolytic index was calculated using the following formula:
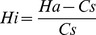
were *Hi* is the hemolytic index, *Ha* the hemolysis area and *Cs* the colony size at the end of the incubation time. Results are presented as means of four independent experiments.

### Western blot analysis

Cultures were grown in bactopeptone medium at 30°C and 250 rpm, and culture supernatants were collected by centrifugation at t2. SDS-PAGE was carried out using 12% acrylamide gels, and silver stained according to Blum *et al.* [42]. Proteins were blotted onto Immun-Blot PVDF membranes (Bio-Rad), and nonspecific binding was blocked with 5% nonfat milk. The HblB (binding) component was detected using monoclonal antibody 2A3 diluted 1∶15 [43], kindly provided by Dr Erwin Märtlbauer (Ludwig-Maximilians-Universität, Munich, Germany). Peroxidase-conjugated AffiniPure Goat-anti-mouse IgG (Jackson Immuno Research Laboratories Inc) were used at 0.8 µg/ml as secondary antibody, and bands were detected using the SuperSignal West Femto Substrate (Pierce) and quantified using ImageJ [41]. After subtraction of the background, the mean gray value for each band was normalized to the intensity of the band in the 407 wild type sample, arbitrarily defined as 1.

### Flow cytometry experiments

Biofilms recovered from glass tubes assays were homogenized by aspirating/pushing ten times through a 26-gauge needle. Planktonic cultures, or homogenized biofilms, were mixed with an equal volume of ice-cold, 0.2 µm-filtered PBS containing 8% formaldehyde, washed with ice-cold PBS and resuspended in TEG (Tris 20 mM EDTA 10 mM glucose 0.5M pH 7.2). Fluorescence was recorded on a Cyflow SL flow cytometer (Partec GmbH, Münster, Germany). YFP fluorescence was measured by using a solid blue-laser emitting at 488 nm, a 620-nm Long Pass Dichroic Mirror and a 590-nm band pass filter (565–615). mCherry fluorescence was measured with a solid yellow-laser emitting at 561 nm combined to a 585-nm band-pass filter. Gating on FSC/SSC was used to discriminate bacteria from the background. For each sample, at least 40,000 gated events were measured. Data were collected with the FlowMax software (Partec GmbH, Münster, Germany) and analysed with the Weasel 2.0 software (WEHI, USA).

## Results

### Spo0A, AbrB and SinI/SinR control biofilm formation and swimming motility

Deletion of *abrB*, *sinI* or *sinI-sinR* did not result in significant changes in growth (figure S1a and S1b). In contrast, the *spo0A* mutant strain grew more slowly (figure S1a), and the *sinR* mutant strain grew poorly (figure S1b). This is not true in *B. anthracis* strain Sterne, where it has previously been described that deletion of *sinR* do not impair bacterial growth [22].

**Figure 1:**
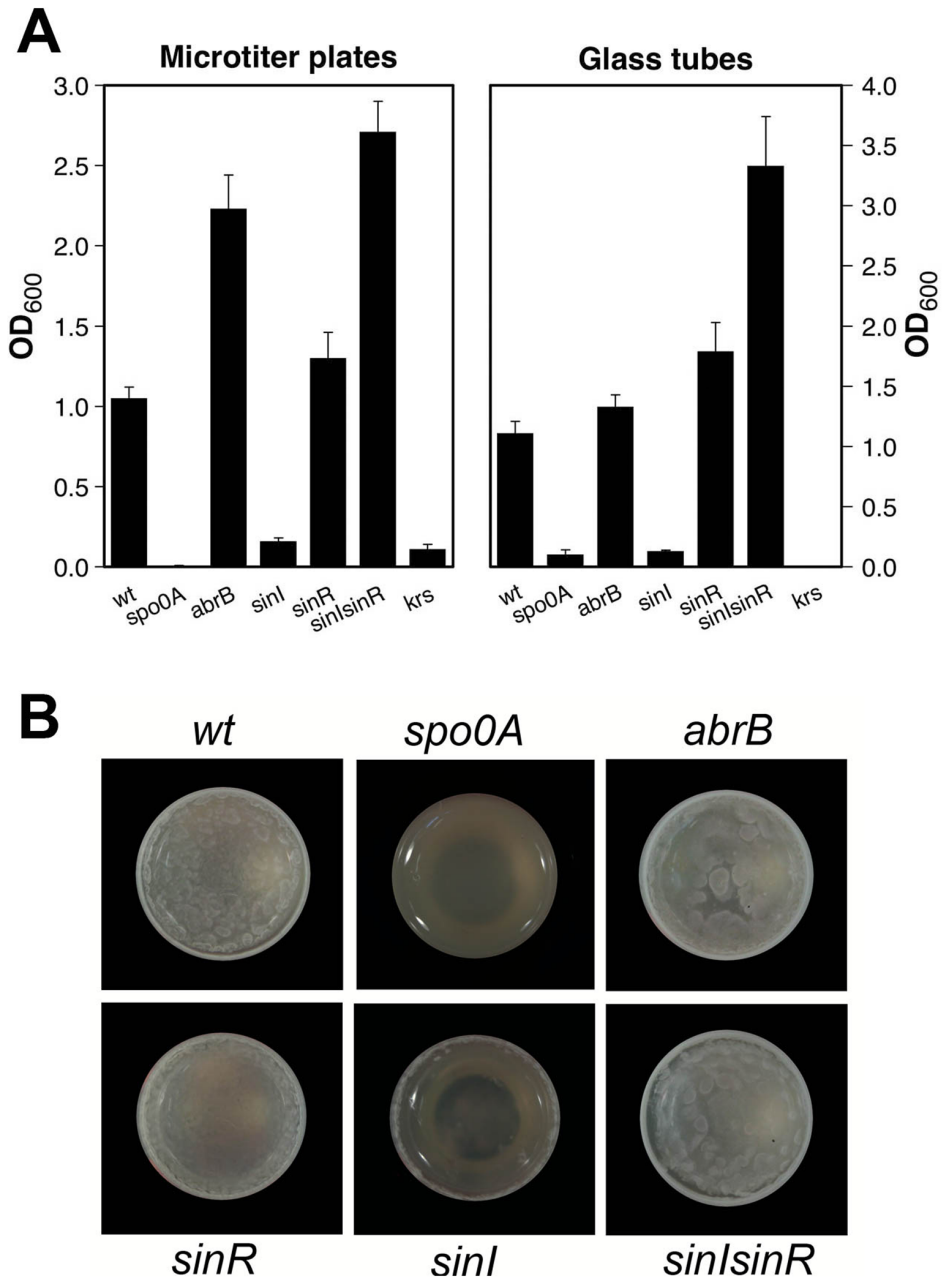
Biofilm formation by mutant strains. Biofilm production by the wild type strain 407 (wt) and by the 407 mutants *spo0A*, *abrB*, *sinI*, *sinR*, *sinI-sinR* and *krsABC*, was assessed in microtiter plates and in glass tubes. A: Bars represent the means of six (microtiter plates) to 12 (glass tubes) replicates from three independent experiments, and error bars represent the standard error of the mean. B: Pictures of the floating pellicles obtained in glass tubes for wild type and mutant strains.

**Figure 2:**
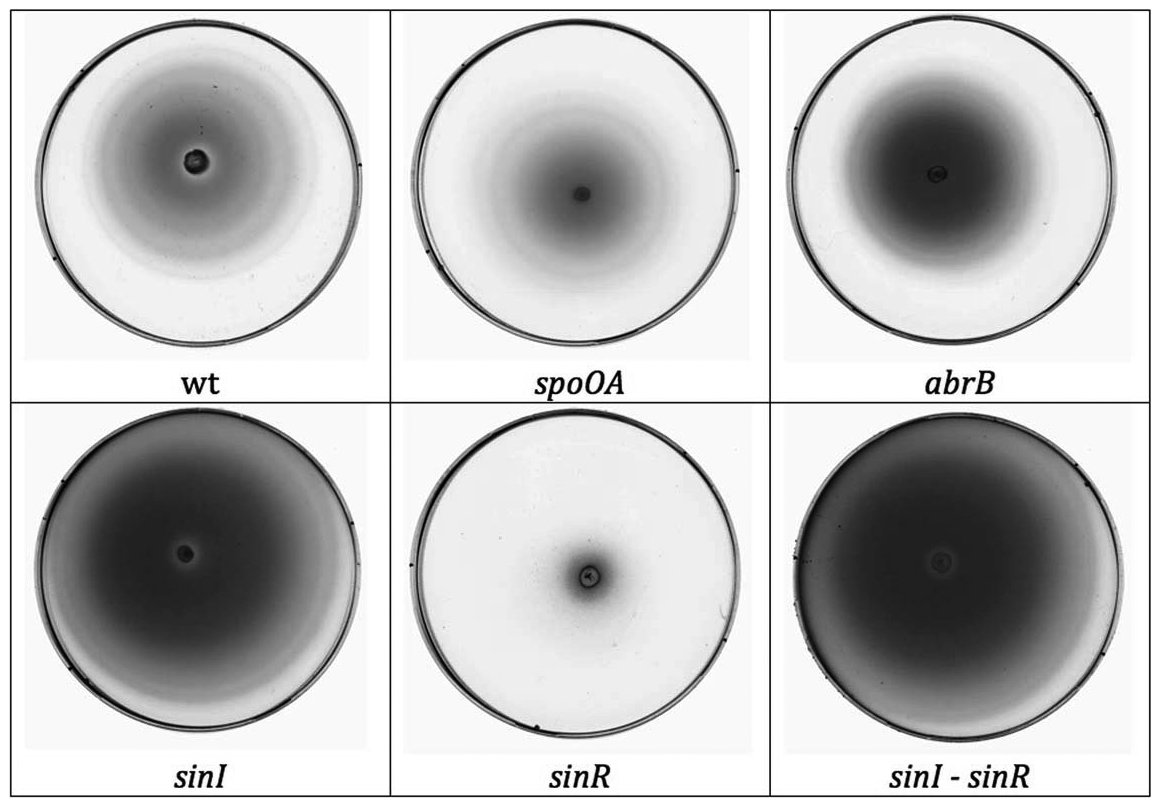
Swimming assays with the mutant strains. Swimming assays were performed on LB agar plates incubated overnight at 30°C, with the wild type strain (wt) and the *spo0A*, *abrB*, *sinI*, *sinR* and *sinIsinR* mutant strains. Each swimming assay was repeated four times in independent experiments, and the results obtained were fairly reproducible. Only one replicate is shown here.

The effect of *spo0A*, *abrB*, *sinI* and *sinR* on biofilm formation and on motility was similar to *B. subtilis* as previously reported. In microtiter plates and in glass tubes, Spo0A promoted biofilm formation while AbrB repressed this phenotype (figure 1A). Both mutants had no effect on swimming motility on 0.3% LB agar plates (figure 2). The *sinI* mutant was highly motile but unable to form biofilms, the *sinR* mutant was non-motile and overproduced bofilms and the *sinI-sinR* mutant was highly motile and overproduced biofilms. The architecture of the wild type strain biofilm, viewed from above in glass tubes, appeared as a thick ring sticking to the tube wall, surrounding a floating pellicle on which protrusions could be seen (figure 1B). This architecture was similar in the *abrB* and the *sinI-sinR* mutants, whereas the *sinR* mutant produced a thick ring and a flat pellicle (figure 1B). In contrast, the *spo0A* mutant produced no ring and no pellicle, and the *sinI* mutant displayed a small ring but no pellicle.

### Microarray analysis of the *sinI*, *sinR* and *sinIsinR* mutants reveals the SinR regulon

We used microarray analysis of strain 407 to identify genes mediating the effects of *sinI* and *sinR* on biofilm formation. A comparative microarray analysis of the *sinR* mutant and of the wild type strain 407, in the early stationary growth phase, revealed 421 repressed genes in the mutant, many of which are associated with the translation machinery (data not shown). The large decrease in the growth rate of strain 407 upon deletion of *sinR* is consistent with this result. To overcome this problem, the transcriptome in the presence and absence of *sinR* was analyzed in a *sinI*-deletion background. Indeed, both *sinI* and *sinI-sinR* mutant strains grew similarly to the wild type strain (figure S1b).

From this analysis, 32 genes appeared to be repressed by SinR two hours after the onset of stationary phase (t2) (Table 2). The *B. cereus* homologue of *B. subtilis sipW* and two *tasA* homologues (BC1278, BC1279 and BC1281), as well as the Hbl enterotoxin genes BC3102 (*hblA* encoding the binding component HblB) and BC3104 (*hblC* encoding the lytic component HblL2) were highly repressed by SinR (Table 2). The *krsEABC* operon (BC2450–BC2453), recently shown to be involved in the biosynthesis and export of a non-ribosomal lipopeptide [30], was also found to be SinR-regulated. Deletion of the *krs* locus abolished the capability to form biofilms either in microtiter plates or in glass tubes (figure 1). The SinR regulon also included as many as nine genes coding for proteins of unknown functions, some of them being strongly repressed by the *sinR* deletion. In addition, two motility genes, encoding the chemotaxis protein CheA (BC1628) and the flagellar basal body rod protein FlgC (BC1642), were slightly repressed by SinR (signal ratio 2.5 and 2, respectively), but this moderate repression probably does not explain the large opposite effect of SinR on motility. Genes possibly involved in detoxification processes (BC2230, BC3076, BC3078, BC4272), in sugar metabolism (BC2960, BC2854, BC3759), in DNA recombination (BC2556) or degradation (BC1072), in peptidoglycan turnover (BC5234), and in energy production (BC3142) were also identified as being regulated by SinR in strain 407 at t2 (table 2). Finally, SinR repressed a gene (BC2410) encoding a PlcR-controlled transcriptional regulator [6].

**Table 2:** Microarray results for the 407 *sinRsinI* mutant compared to the 407 *sinI* mutant.

Locus tag^a^	Gene Product^b^	Ba^c^	SR^d^
BC1278	Signal peptidase I, SipW	GBAA1287	36.8
BC1279	TasA homologue	GBAA1288	26.0
BC1281	Camelysin CalY	GBAA1290	88.6
BC1072	endonuclease/exonuclease/phosphatase family protein	GBAA1075	3.6
BC2556	DNA integration/recombination/invertion protein	NH	2.4
BC3102	Enterotoxin binding component precursor Hbl	NH	8.9
BC3104	Enterotoxin lytic component HblL2	NH	3.9
BC0418	hypothetical protein	NH	9.8
BC1280	hypothetical protein	NH	5.4
BC2409	hypothetical protein	NH	3.9
BC2875	hypothetical protein	NH	4.2
BC3283	hypothetical protein	NH	2.7
BC3290	hypothetical protein	NH	2.2
BC3697	hypothetical protein	NH	3.2
BC4216	hypothetical protein	NH	8.1
BC4259	hypothetical protein	NH	3.1
BC1628	chemotaxis protein CheA	NH	2.5
BC1642	flagellar basal body rod protein FlgC	NH	2.1
BC2230	macrolide-efflux protein MFS-1 family	NH	2.4
BC3076	acetyltransferase	NH	4.4
BC3078	aminoglycoside 3′-phosphotransferase	NH	5.4
BC4272	superoxide dismutase	NH	2.7
BC2854	aldo-keto-oxidoreductase	NH	6.2
BC2960	Glycosyl transferase	NH	2.9
BC3759	6-phospho-beta-glucosidase	NH	3.0
BC2410	TetR family transcriptional regulator	NH	2.2
BC3142	NADPH-dependent oxidoreductase	NH	2.2
BC5234	N-acetylmuramoyl-L-alanine amidase	NH	2.2
BC2450	macrolide-efflux protein MFS-1 family	NH	4.1
BC2451	peptide synthetase	NH	1.3
BC2452	peptide synthetase	NH	1.1
BC2453	peptide synthetase	NH	2.2

Genes are grouped into functional families.

a and b : locus tag and gene product, respectively, according to the annotation of the ATCC14579 genome.

c : homologues in the SinR regulon of *B. anthracis* (Pflughoeft et al., 2011); NH: no homologues.

d : SR, microarray signal ratio, computed as the signal for the *sinIsinR* mutant divided by the signal for the *sinI* mutant.

### *hbl* expression is controlled by SinR

**Figure 3:**
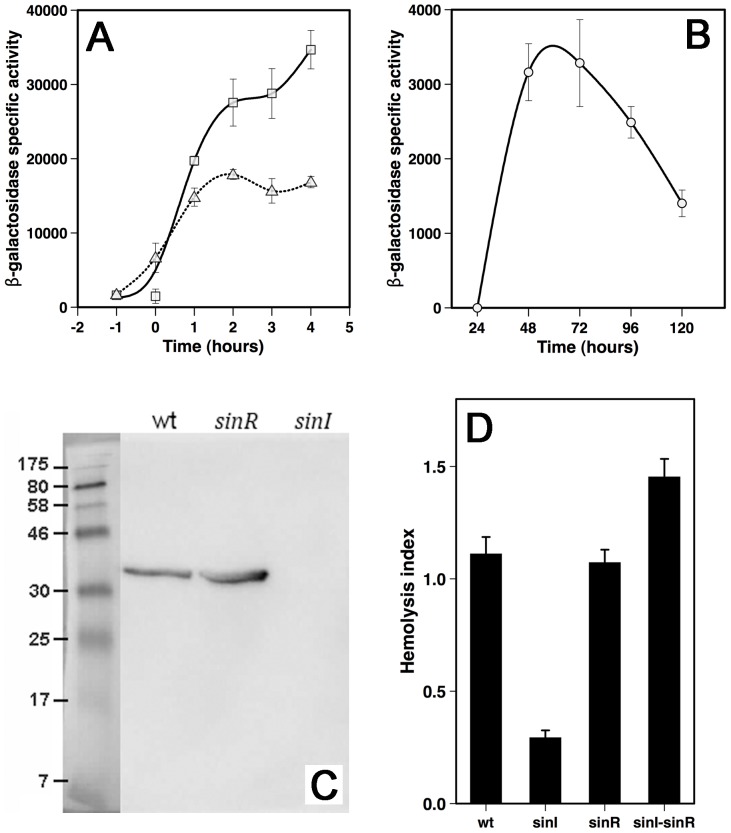
*hbl* expression and hemolytic activity. a and b: transcriptional activity of the *hbl* promoter region (time on the x-axis is relative to entry into stationary phase, and bars represent standard errors of the mean); a: the wt strain (squares) was compared to the *sinI* strain (triangles) in planktonic cultures; b: biofilm glass tube assay. c: Western blot of supernatants from cultures of mutant strains ; band intensities, relative to that for the wild type strain, were: wt: 1.00; *sinR*: 1.64 ; *sinI* : 0.01; d: hemolytic properties of the 407 mutant strains.

Microarray analysis suggested that expression of the Hbl enterotoxin may be controlled by SinR. The Hbl enterotoxin is already known to be controlled by the virulence transcriptional regulator PlcR [44], [45]. To confirm that the *hbl* genes were also under SinR regulation, we used a transcriptional fusion between the *hblC* (BC3104; the first gene of the *hblCDA* operon) promoter region and the *lacZ* gene. As already described [46], expression of *hblC* in the wild type strain increased sharply after t0 and reached a plateau at t2 (figure 3A). Deletion of *sinI* did not abolish *hblC* expression, but reduced it greatly, resulting in a ratio of expression of 2.3 at t4 (figure 3A). The effect of SinR on extracellular HblB (encoded by *hblA*; the third gene of the operon) was then assessed by Western immunoblotting (figure 3C). The amount of extracellular HblB component produced by the *sinR* mutant strain was higher than that of the wild type, whereas no band could be detected in the *sinI* mutant. Hence, SinR repressed HblB production while SinI had the reverse effect. Hbl enterotoxins have hemolytic activity, and these toxins are the major hemolysins acting on sheep blood [47]. Therefore, we studied *sinI*, *sinR* and *sinI*-*sinR* deletion mutants by hemolysis assays on sheep blood agar plates (figure 3D). The hemolytic activity of the *sinI* mutant strain was much lower than that of the wild type strain. In contrast, deletion of *sinR* had no effect on hemolytic activity, and deletion of both *sinI* and *sinR* resulted in higher hemolytic activity.

### *hbl* is expressed in the biofilm produced by strain 407

The co-regulation of biofilm formation and *hbl* expression by SinR suggests that *hbl* could be expressed by strain 407 in biofilm. To test this, we produced biofilms in glass tubes with the wild type strain 407 transformed with pHT304-18ΩP*_hbl_'-lacZ*, the plasmid carrying the P*_hbl_*'-*lacZ* transcriptional fusion, and we followed *hbl* expression in the floating pellicle and the planktonic bacterial population right underneath (figure 3B): *hbl* was expressed in the biofilm, peaking after 48 to 72 hours of culture, when the biofilm has reached its maximal development in glass tubes. This expression, despite decreasing from the peak at 48 h, was sustained and lasted for more than 120 hours, whereas *hbl* expression in planktonic cultures was shut down after t8 (data not shown). Therefore, *B. cereus* biofilms are persistent structures in which the bacteria produce significant amounts of *hbl* over long periods of time.

### *hbl* is expressed by a small subpopulation of biofilm cells

**Figure 4:**
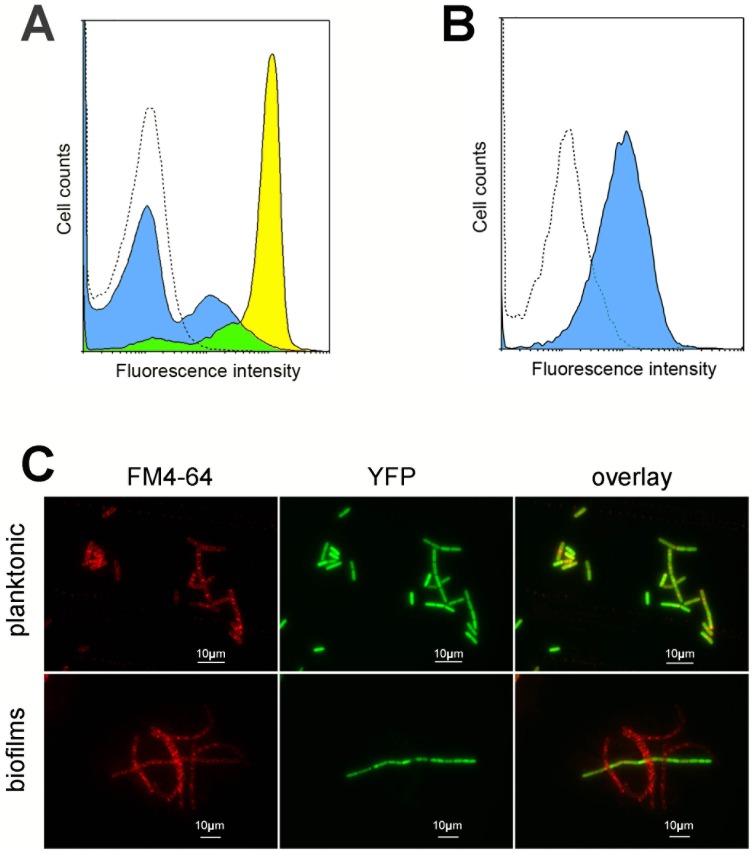
Heterogeneity of *hbl* expression in planktonic cultures and in biofilms. A: Expression from the *hbl* promoter was monitored in planktonic cultures and in biofilms by epifluorescence microscopy through a transcriptional fusion to *yfp*. Cell limits are shown by the membrane stain FM4-64 (red). B: Flow cytometry analysis of bacteria expressing P*_hbl_'-yfp* in planktonic cultures or in biofilms, shown as histogram plot. The blue-filled curve shows biofilm data, the yellow-filled curve shows planktonic cultures data and the unfilled dashed curve shows data from bacteria lacking *yfp*. C: Flow cytometry analysis of bacteria expressing P*_apha3_'-yfp* in biofilms (blue-filled curve) compared to bacteria lacking *yfp* (unfilled dashed curve), shown as histogram plot.

**Figure 5:**
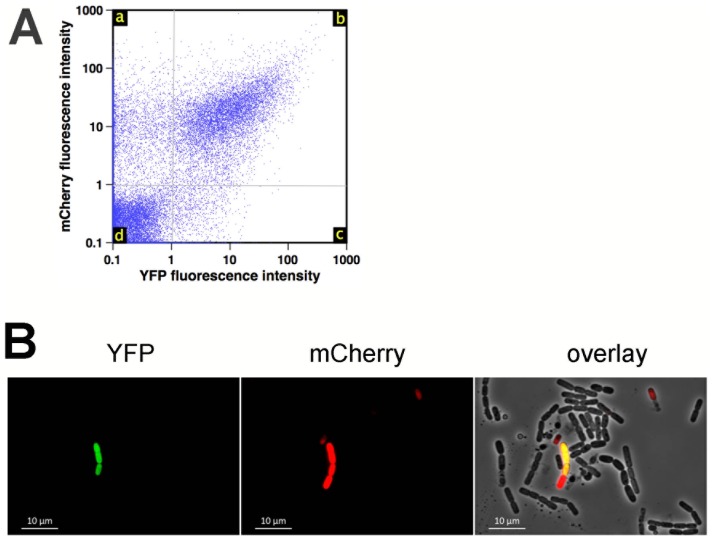
Expression of *hbl* and of *sinI* in biofilms. A: Observation by epifluorescence microscopy of bacteria expressing P*_hbl_'-yfp* (left, in green) and P*_sinI_'-mcherry* (center, in red) in 48 h-old biofilms. An overlay of YFP fluorescence (*hbl* expression), mCherry fluorescence (*sinI* expression) and phase contrast microscopy is shown on the right. B: Flow cytometry analysis of bacteria expressing P*_hbl_'-yfp* and P*_sinI_'-mcherry* in 48 h-old biofilms, shown as dot-plot. While 72% of the bacteria do not express *hbl* nor *sinI* (quadrant d), 15% of the cells which express *hbl* also express *sinI* (quadrant b), and 12% of the bacteria express *sinI* but not *hbl* (quadrant a).

To determine the proportion of *B. cereus* cells expressing *hbl* enterotoxin genes, we used a transcriptional fusion between the promoter region of *hbl* and the yellow fluorescence protein gene *yfp*. Strain 407 expressing *yfp* under the control of the *hbl* promoter was grown in planktonic cultures and in biofilms, and samples were harvested when *hbl* expression reached a plateau or was maximal as determined with *lacZ* fusions (figure 3A and 3B: t2 for planktonic cultures and 48 h for biofilms). As shown by flow cytometry, the *hbl* promoter was active in 90% of bacteria in planktonic cultures in early stationary phase and in 33% of bacteria in homogenized 48 h-old biofilms (figure 4A). In contrast, the *apha3* constitutive promoter was active in 88% of bacteria in 48 h-old biofilms (figure 4B), showing that the heterogeneity of *hbl* expression in biofilms was not consecutive to non-viable bacterial cells or to plasmid loss in this culture condition. In addition, colonies recovered from 48 h-old biofilms formed with the 407 strain carrying the pHT304-18ΩP*_hbl_'-yfp*, and transfered to LB- and erythromycin-LB plates, were 100%±0.0 resistant to erythromycin, the pHT304 resistance marker (3 independent experiments). Flow cytometry also revealed that *hbl* was on average transcribed at a lower level in the biofilm than in planktonic cultures (figure 4A). These results are supported by epifluorescence microscopy, which showed that almost all bacteria in planktonic cultures expressed *hbl* whereas only a few expressed it in biofilms (figure 4C). By using a plasmid carrying both the P*_hbl_'*-*yfp* and the *P_sinI_'-mcherry* transcriptional fusions, we have monitored the expression of *hbl* and *sinI* in the same cells in 48 h-old biofilms. We found that 16% of the bacteria expressed *hbl* (figure 5A), which is in the same range as our previous results. Furthermore, flow cytometry and microscopy observation revealed that almost all bacteria expressing *hbl* also expressed *sinI* (figure 5A and 5B). In addition, 12% of the bacteria expressed *sinI* but not *hbl*.

## Discussion

We deleted from *B. thuringiensis* the genes encoding Spo0A, AbrB and SinR, which are regulators of the transition phase of growth. These regulators were previously shown to control biofilm formation and swarming motility in *B. subtilis*. We report here that, in *B. thuringiensis*, SinR represses biofilm formation and is required for swimming motility, whereas SinI has the reverse effect. Consequently, the SinI/SinR antirepressor/repressor pair is likely to act as a switch between biofilm formation and swimming motility, as it does in *B. subtilis* between biofilm formation and swarming motility [48]. In addition, Spo0A is required in *B. thuringiensis* for biofilm formation and AbrB represses this phenotype, and neither of these regulators affects motility. These findings suggest that the regulation of biofilm formation and of motility by Spo0A, AbrB, and SinI/SinR show similarities in *B. cereus* and in *B. subtilis*. Similarities between the two species for control of biofilm formation is supported by the presence of the *sipW*-*tasA* operon in their respective SinR regulons. The *sipW*-*tasA* operon was shown to be required for the production of the proteic component of the biofilm matrix in *B. subtilis*, and to be directly controlled by SinR in *B. anthracis* [22].

However, within the 32 genes included in the *B. thuringiensis* SinR regulon, only *sipW* and *tasA* are shared with the *B. subtilis* SinR regulon reported previously [15]. *B. thuringiensis* and *B. cereus* display a chromosomal conserved locus (genes BC5267 to BC5278 in *B. cereus* strain ATCC14579) similar to the *epsAO* locus which in *B. subtilis* is involved in the biosynthesis of the exopolysaccharide component of the biofilm matrix. A 120 bp antitermination RNA element named EAR is found, in *B. subtilis*, exclusivel*y* only in the *epsAO* locus [49], and a corresponding element is predicted to be present in the BC5267–BC5278 locus, consistent with these loci being homologous. But while the *B. subtilis epsAO* genes are repressed by SinR, the *B. thuringiensis* BC5267–BC5278 orthologs are not.

Conversely, the *B. thuringiensis* - but not the *B. subtilis* - SinR regulon includes genes required for the production of a lipopeptide. This lipopeptide, kurstakin, is required for biofilm formation. In *B. subtilis*, production of the lipopeptide surfactin, also important for biofilm formation [16], is controlled by the two-component system ComA-ComP [50]. This two-component system is not present in bacteria of the *B. cereus* group, but it was recently shown that kurstakin production is activated by the NprR cell-cell communication system during stationary phase [30]. In addition to its role in biofilm formation, kurstakin was shown to be required for bacterial survival in the host cadaver [50].

Our transcriptional analysis also revealed that SinR repressed the *hbl* enterotoxin genes. The transcription of *hbl* genes is promoted by the virulence regulator PlcR [44], [45]. The products of these genes are active against the rabbit intestine [51] and may be responsible for the diarrheal symptoms associated with *B. cereus*-dependent gastroenteritis [7]. Transcriptional fusion experiments confirmed that SinI/SinR controls *hbl* transcription, and Western blotting and hemolysis assays also showed that SinI/SinR regulates the production of Hbl enterotoxin components. The SinR regulon includes another gene controlled by PlcR: BC2410. The product of BC2410 is a transcriptional regulator, the targets of which are still unknown, but which might be involved in bacterial pathogenesis [6]. Therefore, SinR in *B. cereus* controls virulence factors that are part of the PlcR regulon, in addition to biofilm formation and motility. These findings suggest that inactivation of SinR by SinI both serve to trigger biofilm formation and to enhance enterotoxin production.

We assessed the expression of *hbl* in biofilms using *lacZ* fusions. We found that this expression was sustained and lasted for more than 48 h but was moderate as compared to the strong expression of *hbl* in planktonic cultures. We determined *hbl* expression at the cell level using *yfp* fusions: most of the bacteria in planktonic cultures expressed *hbl*, while only a small subpopulation of cells expressed it in biofilms. This heterogeneity in the expression of *hbl* in biofilms is likely to be a consequence of the heterogeneity in the expression of *sinI*, since we found that bacteria expressing *hbl* also expressed *sinI*. SinI-dependent heterogeneity of genes expression in biofilms has been described in *B. subtilis*, where the *sipW-tasA* operon is expressed in the same subpopulation as *sinI*, whereas *sinR* is expressed in the whole biofilm bacterial population [52].

The *B. thuringiensis* SinR regulon shares only 4 genes with the *B. anthracis* SinR regulon as determined previously [22] : the homologue of *B. subtilis sipW* and two homologues of *tasA*, and a gene encoding an endonuclease. The 28 other genes of the *B. thuringiensis* SinR regulon have not been identified as components of the *B. anthracis* SinR regulon, indicating possible differences between these two species for the role of SinR. More specifically, the *inhA1* gene, encoding a metalloprotease, has previously been reported to be SinR-dependent in *B. anthracis* [22]. This gene is also present in *B. thuringiensis* and *B. cereus*, were it is likely to play an important role in the bacterial pathogenesis [53], [54]. Although *inhA1* was previously reported to be controlled by Spo0A, AbrB and SinR in the 407 strain [55], we do not confirm, by microarray analysis, the role of SinR in the regulation of *inhA1* transcription. However, the previous study used an overexpression of *sinI* or of *sinR* on high copy plasmids to investigate the role of SinR in the control of *inhA1*, which might have introduced bias in that study.

The *sinI-sinR* mutant produced more biofilm and was more hemolytic than the *sinR* mutant, which was unexpected. If the function of SinI is only antagonizes SinR, then *sinI-sinR* and *sinR* mutants should have similar phenotypes, and give similar results. The observed difference could suggest that SinI also acts on another regulator, different from SinR. In *B. subtilis*, biofilm formation is stimulated by SlrR, a paralogue of SinR [21], [56], and SlrR interacts with SlrA, a paralogue of SinI [57]. Possibly, SinI in *B. thuringiensis* inactivates both SinR and a putative paralogue of SinR.

In the present work, we have shown that *B. thuringiensis* and *B. subtilis* have similarities and differences in the control of biofilm formation. The SinR regulon of the two species have only two genes in common under the conditions studied. In *B. thuringiensis*, the SinR regulon includes the *hbl* gene and the *krsEABC* locus, which are involved in the interaction of the bacterium with its host. The Hbl toxin complex is cytotoxic and causes damage to the intestinal tract [7], and kurstakin is required for biofilm formation and for bacterial survival in the host cadaver. Therefore, SinR co-regulates both biofilm formation and part of the infectious process in *B. thuringiensis*, which makes sense if we consider that this pathogen can settle in heterologous biofilms [58], and therefore potentially integrate into the biofilm microbiota lining the host intestinal epithelium. In this biofilm, toxins could be delivered directly to their target tissue.

## Supporting Information

Figure S1 **Growth curves.** The various strains were grown in LB medium at 37°C and 175 rpm. The OD was measured at 600 nm and plotted against time. a: wild-type strain (black circle), *spo0A* mutant (white circle), *abrB* mutant (white triangle). b: wild-type strain (black circle), *sinR* mutant (white circle), *sinI* mutant (white triangle), *sinI—sinR* mutant (white inverted triangle).
(DOC)

Table S1 **Strains used in this study.**
(DOC)

Table S2 **Correspondence between the locus tags in the ATCC14579 and in the 407 strains.** No ortholog annotated in *B. thuringiensis* 407 and no DNA with similarity to the indicated *B. cereus* ATCC 14579 ORF present in the *B. thuringiensis* 407 genome. Could potentially be a result of missing data in the draft *B. thuringiensis* 407 sequence.
(DOC)
