# Correction: SinR Controls Enterotoxin Expression in *Bacillus thuringiensis* Biofilms

**DOI:** 10.1371/journal.pone.0096707

**Published:** 2014-04-25

**Authors:** The PLOS ONE Staff

The figure legends for Figure 4 and Figure 5 contain misnumbered subfigures in the PDF and XML versions of the article. Please see these figures and their corrected figure legends below.

**Figure 4:**
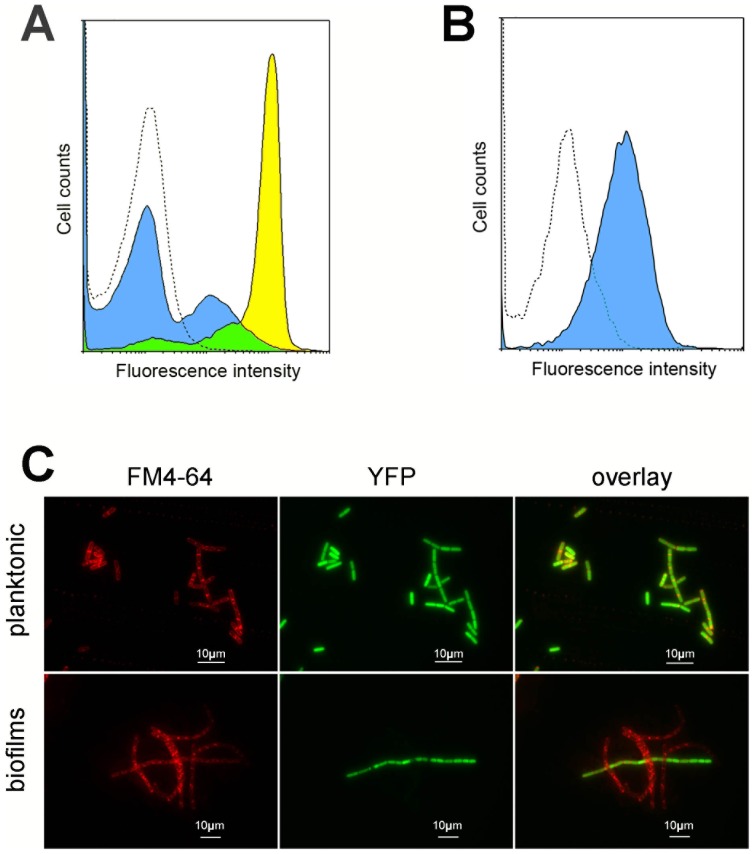
Heterogeneity of *hbl* expression in planktonic cultures and in biofilms. A: Flow cytometry analysis of bacteria expressing P*_hbl_'-yfp* in planktonic cultures or in biofilms, shown as histogram plot. The blue-filled curve shows biofilm data, the yellow-filled curve shows planktonic cultures data and the unfilled dashed curve shows data from bacteria lacking *yfp*. B: Flow cytometry analysis of bacteria expressing P*_apha3_'-yfp* in biofilms (blue-filled curve) compared to bacteria lacking *yfp* (unfilled dashed curve), shown as histogram plot. C: Expression from the *hbl* promoter was monitored in planktonic cultures and in biofilms by epifluorescence microscopy through a transcriptional fusion to *yfp*. Cell limits are shown by the membrane stain FM4-64 (red).

**Figure 5:**
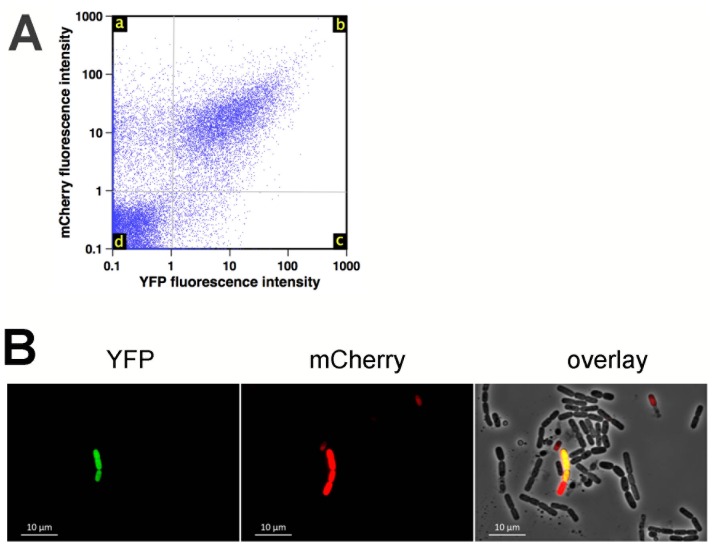
Expression of hbl and of sinI in biofilms. A: Flow cytometry analysis of bacteria expressing P*_hbl_'-yfp* and P*_sinI_'-mcherry* in 48 h-old biofilms, shown as dot-plot. While 72% of the bacteria do not express *hbl* nor *sinI* (quadrant d), 15% of the cells which express *hbl* also express *sinI* (quadrant b), and 12% of the bacteria express *sinI* but not*hbl* (quadrant a). B: Observation by epifluorescence microscopy of bacteria expressing P*_hbl_'-yfp* (left, in green) and P*_sinI_'-mcherry* (center, in red) in 48 h-old biofilms. An overlay of YFP fluorescence (*hbl* expression), mCherry fluorescence (*sinI* expression) and phase contrast microscopy is shown on the right.

## References

[1] Fagerlund A, Dubois T, Økstad O-A, Verplaetse E, Gilois N, et al (2014) SinR Controls Enterotoxin Expression in *Bacillus thuringiensis* Biofilms. PLoS ONE 9(1): e87532 doi:10.1371/journal.pone.0087532 24498128 10.1371/journal.pone.0087532 PMC3909190

