# Supplementary material for: SinR Controls Enterotoxin Expression in Bacillus thuringiensis Biofilms
Source: PLoS One. 2014 Jan 31;9(1):e87532. doi: 10.1371/journal.pone.0087532 (PMC3909190; doi:10.1371/journal.pone.0087532)
Supplement: Figure S1 — Growth curves. The various strains were grown in LB medium at 37°C and 175 rpm. The OD was measured at 600 nm and plotted against time. a: wild-type strain (black circle), spo0A mutant (white circle), abrB mutant (white triangle). b: wild-type strain (black circle), sinR mutant (white circle), sinI mutant (white triangle), sinI—sinR mutant (white inverted triangle). (DOC) [file pone.0087532.s001.doc]

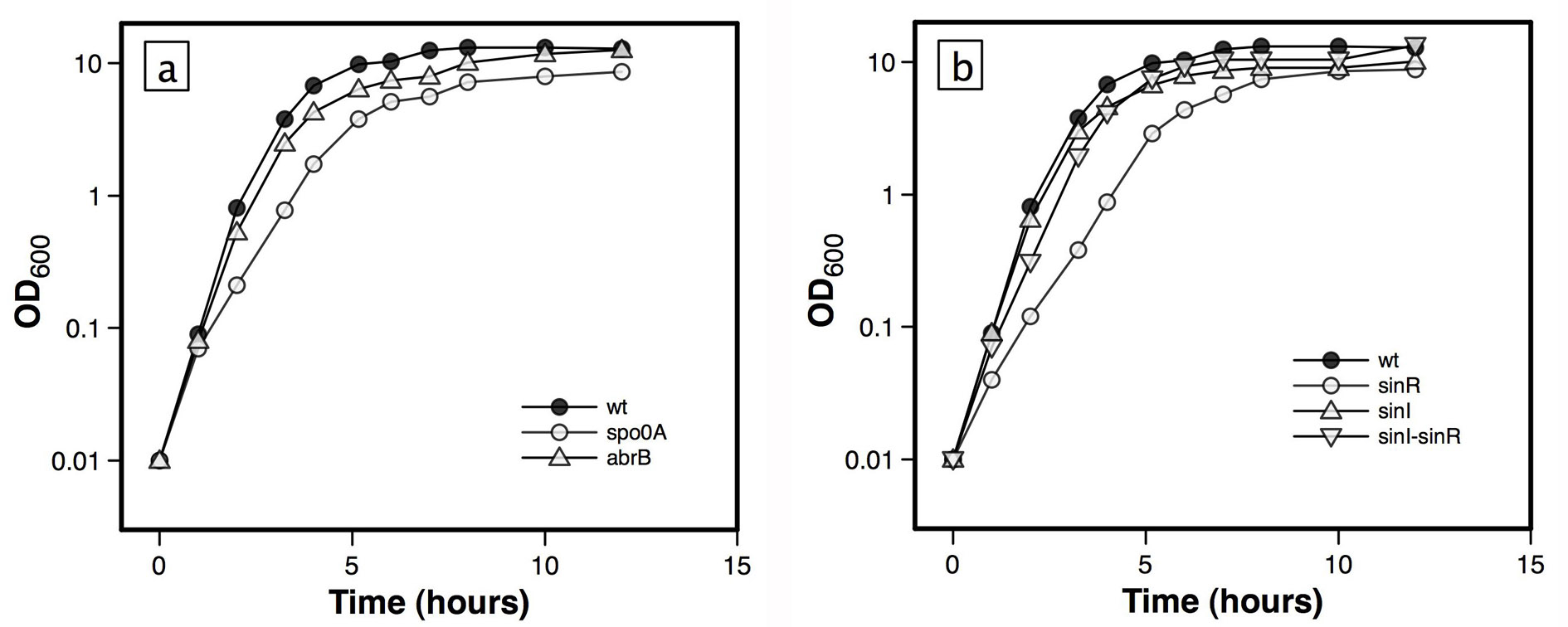


Figure S1 : Growth curves

The various strains were grown in LB medium at 37°C and 175rpm. The OD was measured at 600nm and plotted against time.

a: wild-type strain (black circle), *spo0A* mutant (white circle), *abrB* mutant (white triangle).

b: wild-type strain (black circle), *sinR* mutant (white circle), *sinI* mutant (white triangle), *sinI—sinR* mutant (white inverted triangle).
