# Supplementary material for: SinR Controls Enterotoxin Expression in Bacillus thuringiensis Biofilms
Source: PLoS One. 2014 Jan 31;9(1):e87532. doi: 10.1371/journal.pone.0087532 (PMC3909190; doi:10.1371/journal.pone.0087532)
Supplement: Table S1 — Strains used in this study. (DOC) [file pone.0087532.s002.doc]

| Strain | description | reference/source |
| --- | --- | --- |
| *E. coli* TG1 | General cloning strain | Stratagene |
| *E. coli* ET 12567 | Methylation defective strain *(dam-13::Tn9, dcm-6, hsdkf)* |  |
| 407 wt | *B. thuringiensis* strain 407 Cryˉ |  |
| 407 *spo0A* | Strain 407 were the *spo0A* gene is deleted by insertion of a kanamycin resistance cassette |  |
| 407 *krs* | Strain 407 were the *krs* operon is deleted by insertion of a spectinomycin resistance cassette |  |
| 407 *abrB* | Strain 407 were the *abrB* gene is deleted by insertion of a kanamycin resistance cassette | this study |
| 407 *sinI* | Strain 407 were the *sinI* gene is deleted by insertion of a tetracyclin resistance cassette | this study |
| 407 *sinR* | Strain 407 were the *sinR* gene is deleted by insertion of a tetracyclin resistance cassette | this study |
| 407 *sinI sinR* | Strain 407 were the *sinI* and *sinR* genes are deleted by insertion of a tetracyclin resistance cassette | this study |

Table S1: strains used in this study

References

1. MacNeil DJ, Gewain KM, Ruby CL, Dezeny G, Gibbons PH, et al. (1992) Analysis of *Streptomyces avermitilis* genes required for avermectin biosynthesis utilizing a novel integration vector. Gene 111: 61-68.

2. Lereclus D, Arantes O, Chaufaux J, Lecadet M (1989) Transformation and expression of a cloned delta-endotoxin gene in *Bacillus thuringiensis*. FEMS Microbiology Letters 51: 211-217.

3. Lereclus D, Agaisse H, Gominet M, Chaufaux J (1995) Overproduction of encapsulated insecticidal crystal proteins in a *Bacillus thuringiensis spo0A* mutant. Biotechnology (N Y) 13: 69-71.

4. Dubois T, Faegri K, Perchat S, Lemy C, Buisson C, et al. (2012) Necrotrophism is a quorum-sensing-regulated lifestyle in *Bacillus thuringiensis*. PLoS Pathog 8: e1002629.
