# Supplementary material for: SinR Controls Enterotoxin Expression in Bacillus thuringiensis Biofilms
Source: PLoS One. 2014 Jan 31;9(1):e87532. doi: 10.1371/journal.pone.0087532 (PMC3909190; doi:10.1371/journal.pone.0087532)
Supplement: Table S2 — Correspondence between the locus tags in the ATCC14579 and in the 407 strains. No ortholog annotated in B. thuringiensis 407 and no DNA with similarity to the indicated B. cereus ATCC 14579 ORF present in the B. thuringiensis 407 genome. Could potentially be a result of missing data in the draft B. thuringiensis 407 sequence. (DOC) [file pone.0087532.s003.doc]

| **Locus tag in *Bacillus cereus* ATCC 14579** | **Corresponding locus tag in *Bacillus thuringiensis* 407** |
| --- | --- |
| BC1278 | bthur0002_11880 |
| BC1279 | bthur0002_11890 |
| BC1281 | bthur0002_11910 |
| BC1072 | bthur0002_10050 |
| BC2556 | bthur0002_23910 |
| BC3102 | bthur0002_28440 |
| BC3104 | bthur0002_28460 |
| BC0418 | No locus tag in Bt 407 - ORF is not annotated in genome sequence, but is present in the Bt 407 genome |
| BC1280 | bthur0002_11900 |
| BC2409 | bthur0002_22700 |
| BC2875 | NA^¶^ |
| BC3283 | NA^¶^ |
| BC3290 | NA^¶^ |
| BC3697 | NA^¶^ |
| BC4216 | bthur0002_40650 |
| BC4259 | bthur0002_41130 |
| BC1628 | bthur0002_15380 |
| BC1642 | bthur0002_15520 |
| BC2230 | bthur0002_21030 |
| BC3076 | bthur0002_28710 |
| BC3078 | bthur0002_28740 |
| BC4272 | bthur0002_41260 |
| BC2854 | bthur0002_26270 |
| BC2960 | bthur0002_27450 |
| BC3759 | bthur0002_34830 |
| BC2410 | bthur0002_22720 |
| BC3142 | bthur0002_29110 |
| BC5234 | bthur0002_50980 |
| BC2450 | bthur0002_23000 |
| BC2451 | bthur0002_23010 |
| BC2452 | bthur0002_23010 |
| BC2453 | bthur0002_23020 |

^¶^ No ortholog annotated in *B. thuringiensis* 407 and no DNA with similarity to the indicated *B. cereus* ATCC 14579 ORF present in the *B. thuringiensis* 407 genome. Could potentially be a result of missing data in the draft *B. thuringiensis* 407 sequence.

Table S2 : Correspondence between the locus tags in the ATCC14579 and in the 407 strains. ^¶^ No ortholog annotated in *B. thuringiensis* 407 and no DNA with similarity to the indicated *B. cereus* ATCC 14579 ORF present in the *B. thuringiensis* 407 genome. Could potentially be a result of missing data in the draft *B. thuringiensis* 407 sequence.
